# 

**Published:** 1892-01

**Authors:** 


### CONTENTS:

Article I.—Minutes of the Twenty-third Annual Session of the Southern Dental Association. - - - - - 385

" II.—Pental ($C_5H_{10}$) as an Anæsthetic. By Prof. Ludwig Hollaender. - - - - - - - - 401

" III.—Origin of Pus. By J. B. Patrick, - - - 410

" IV.—Anti-Extraction. By Wm. N. Morrison, D. D. S. - 417

### COMMUNICATIONS.

Post Graduate Dental Association. - - - - - - 421

### EDITORIAL.

The Maryland Dental Law. - - - - - - 421

### MONTHLY SUMMARY.

Clinical Nights at the Societies. - - - - - - - 425

Pyoktanin as an Antiseptic. - - - - - - - 426

Eruption of the Deciduous Teeth, and General Symptoms. - - 427

Death from Tooth Sepsis. - - - - - - - - 428

Flowers of the Red Rose for Chronic Diarrhœa. - - - 428

Trigeminal Neuralgia and Iodide of Potassium. - - - 429

Dark Spots on the Teeth. - - - - - - - 429

A New Society, - - - - - - - - 429

Thirty-six Teeth in a Set. - - - - - - - 430

A Safe Local Anæsthetic. - - - - - - - 430

Cleansing the Hands. - - - - - - - 430

Temporary Sets. - - - - - - - - 431

The Human Breath. - - - - - - - 431

Obtunding the Bite. - - - - - - - 431

Mortality of the Human Race, - - - - - - 432

An Interesting Reproduction. - - - - - - 432

Profuse Hemorrhage. - - - - - - - 432

